# Advanced Oxidation Processes in Triton X-100 and Wash-up Liquid Removal from Wastewater Using Modified TiO_2_/Al_2_O_3_ Photocatalysts

**DOI:** 10.1007/s11270-012-1237-y

**Published:** 2012-06-29

**Authors:** Bożena Czech, Wiesława Ćwikła-Bundyra

**Affiliations:** Department of Environmental Chemistry, Faculty of Chemistry, Maria Curie-Sklodowska University, Pl. M. Curie-Sklodowskiej 3, 20-031 Lublin, Poland

**Keywords:** Photocatalysis, TiO_2_/Al_2_O_3_, AOP, Surfactants, H_2_O_2_

## Abstract

Photocatalytic methods were applied to remove the recalcitrant or toxic pollutants from the water. The two models of wastewater containing either non-ionic surfactant Triton X-100 or commercially available wash-up liquid were tested in a self-constructed band reactor during the laboratory studies. The photocatalyst, being typed TiO_2_, was supported by porous Al_2_O_3_ and modified by the addition of Cu, Fe, Zn, Ni, Mo or Co. The photocatalysts were characterised by N_2_ adsorption–desorption, XRF, XRD, SEM-EDX, Raman and UV–Vis spectroscopy. All catalysts were efficient in the photocatalytic oxidation of surfactants, and they enabled at least 85 % COD reduction. TiO_2_/Al_2_O_3_ photocatalysts modified by the transition metals were efficient only for more complicated compositions of surfactants. The effect of H_2_O_2_ (0.01 vol.%) addition was also examined and compared with a type of compound and catalyst used—in this case a positive effect for Triton X-100 was only observed over the photocatalyst modified by Ni. When it comes to the wash-up liquid photoremoval, all studied photocatalysts seem to be slightly influenced by H_2_O_2_ addition. It was also observed that it is not economically justified to conduct such treatment for more than 2 h.

## Introduction

Surfactants, because of their specific features, are widely used in the households as detergents or care products and in various industries: pesticides, chemicals, textiles or pharmaceuticals. They are common pollutants in the wastewater. It has been confirmed that generally non-ionic surfactants (at lethal concentrations depending on various tests from 0.0025 to 300 mg/dm^3^) are more toxic than others (anionic: 0.3 to 200 mg/dm^3^). Periods of long acclimation are required during the conventional treatment, and they typically result in a quite incomplete degradation (Liwarska-Bizukojc et al. 2005). Finally, introduced into water ecosystem, some of the pollutants such as alkylphenyl polyethoxylate-type non-ionic surfactants, e.g. Triton X-100 (TX), are suspected to be the endocrine disrupters in aquatic organisms (Saien et al. 2011). In order for the surfactants to be removed, the heterogeneous photocatalysis, as a one of the advanced oxidation processes (AOPs), can be applied successfully (Arslan-Alaton and Erdinc 2006; Fabbri et al. 2009). The observed progress in the field of AOPs has made them as alternatives or as a complement to conventional wastewater treatment. The main advantage of AOPs is that they can be used both independently or as pre-treatment technique for improving the biodegradability and efficiency of further treatments (Ioannou et al. 2011).

TiO_2_ is a widely tested photocatalyst in the studies concerning the photocatalytic removal of contaminants (Ollis et al. 1991; Friedmann et al. 2010). However, the recycling and recovery of typically used TiO_2_ powder photocatalysts in industry are quite troublesome and uneconomical, which becomes the main disadvantage of using the suspended systems. Therefore, many efforts are being made to immobilize TiO_2_ and construct reactors with an immobilized active phase (Chong et al. 2010).

Nowadays, photocatalytic studies are being conducted in three main areas: catalyst modifications, reactor construction and process modification (Lin et al. 2010). The catalyst modifications include metal and non-metal doping (Janus et al. 2009; Yalcin et al. 2010). Transition metal ion doping has been suggested to increase the response to solar spectrum over 380 nm and to develop the photocatalytic activity by introducing the defects into TiO_2_ lattice and reducing e^−^/h^+^ pair recombination. The result of the above has been the increased rate of ^*^OH formation. The photocatalytic processes and their efficiency can also be enhanced by adding the auxiliary electron acceptors, such as H_2_O_2_ and O_3_ (Friedmann et al. 2010), but the results are not obvious and depend on many factors. H_2_O_2_ is one of the cheapest oxidants, and in the presence of UV irradiation at 254 nm, its photolysis into 2 ^*^OH has been observed. However, the addition of too high H_2_O_2_ concentration hinders the process, since the excess of H_2_O_2_ captures the radicals (Daneshvar et al. 2004).

The double aptitude of the photocatalyst to simultaneously adsorb reactants and to absorb photons efficiently is one of the primary factors affecting the photocatalysis. It has been observed that due to the pre-adsorption of reactants on the surface of TiO_2_ during the photocatalytic reaction, the process of electron transfer is more efficient (Popa et al. 2010). The supported porous catalysts with higher surface area are often being chosen, since, apart from easier disposal, they ensure a high density of active centers for photocatalytic reactions and an enhanced light harvesting because of light reflection and scattering by the pores (Herrmann 2010).

Since all strategies, surface deposition of transition metals on photocatalyst, porous materials as the support for TiO_2_ and H_2_O_2_ as additional source of ^*^OH, can enhance photooxidation, it was proposed to study the synergistic effect of both support (Al_2_O_3_), H_2_O_2_ addition and dopant (Cu, Zn, Fe, Co, Ni, Mo) on the UV photocatalytic activity of TiO_2_. In the present investigation, we carried out a series of studies aiming for the determination of the catalytic activity of Al_2_O_3_ supported and modified TiO_2_ photocatalysts in the removal of the pollutants from wastewater. As photocatalysts TiO_2_/Al_2_O_3_ modified by Cu, Zn, Ni, Fe, Co or Mo were used. These dopants were chosen because of their ability to increase the solar spectrum response towards Vis or enhance the UV photoactivity. The aim of the research was also to investigate the role of dopant on UV photocatalytic activity of studied catalysts. The photocatalytic oxidation of TX, a non-ionic surfactant, and a mixture of common surfactants, containing both non-ionic and anionic compounds available as commercial wash-up liquid (WL) were investigated. The effectiveness of the removal was measured in the following configurations: TiO_2_/UV/O_2_ and TiO_2_/UV/O_2_/H_2_O_2_. TX was chosen because of its main features: it is a widely used non-ionic surfactant and it can be considered as an endocrine disrupting compound in the aquatic environment. Typically used in households, wash-up liquid was used to examine the photoremoval of commercially available products that are not analytical grade.

## Material and Methods

### Catalysts Preparation

All the chemicals used for the preparation of the photocatalysts were analytical grade and used without further purification. The modified TiO_2_ catalysts supported by γ–Al_2_O_3_ (INS Pulawy, Poland) were chosen for the studies. The catalysts were prepared using the classical impregnation method (CIM) according to the procedure described in (Pasieczna-Patkowska et al. 2010). For the impregnation of TiO_2_/Al_2_O_3_ (sample TiO_2_) were used 5 wt.% aqueous solutions of nitrates: zinc(II), copper(II), cobalt(II), iron(III), nickel(II) and molybdenum salt (NH_4_)_6_Mo_7_O_24_•4H_2_O (POCH Gliwice, Poland) resulted in samples CIM-Zn, CIM-Cu, CIM-Co, CIM-Fe, CIM-Ni and CIM-Mo, respectively. The effect of doping was compared with the sample TiO_2_ as reference.

### Photoreactor and Photocatalytic Studies

The experiments concerning the photocatalytic degradation of organics in the wastewater were conducted in a band reactor of our own construction described in a previous paper (Czech and Cwikla-Bundyra 2007) applying the procedure described in the already mentioned paper (Pasieczna-Patkowska et al. 2010). The band reactor is equipped with the UV lamp (254 nm, 50 Hz) with a light intensity of 1.5–2.2 mW/cm^2^, measured by the Radiometer VLX254 (Vilber Lourmat, 254 nm). The employed UV lamp emitted light of more than 95 % within the UV light wavelength.

Triton X-100 (POCH Gliwice, Poland) and a commercially available wash-up liquid (“Ludwik”, Inco Veritas, Poland) were chosen to be the model contaminants. The solutions were prepared so as to have the COD value of ca. 3,000 mg O_2_/dm^3^, which was the concentration of 1.3 × 10^−3^ vol.% for TX, and the concentration of wash-up liquid was 0.65 × 10^−3^ vol.%. The wash-up liquid contained: 5–15 % anionic, <5 % non-ionic and <5 % amphoteric surfactants and many others (e.g. oxyethylene fatty alcohols, amine oxides, EDTA, acetic acid, polyethylene glycol, 2-bromo-2-nitropropane-1,3-diol and inorganic additions). Hydrogen peroxide (0.01 vol.%) (Standard, Lublin, Poland) was also applied.

### Catalysts Examination

The total surface areas of the catalysts were determined on the basis of nitrogen adsorption at liquid nitrogen temperature using the BET method in a volumetric apparatus ensuring a vacuum of at least 2 × 10^−6^ kPa (AUTOSORB-1CMS, Quantachrome Instruments, USA). The phase composition diagram of the catalysts was determined by the X-ray diffraction (XRD), (HZG-4, Carl Zeiss Jena). Raman spectroscopy was applied to show the crystallographic orientation of a sample **(**inVia Reflex, Renishaw, UK) and the UV–Vis spectroscopy was applied to characterize the absorbance spectra of the photocatalysts. The sample morphology was observed in the scanning electron microscope (SEM, Quanta 3D FEG), equipped with an energy dispersive X-ray detector (EDX), which was used for the determination of the surface elemental composition. The physicochemical properties of the studied catalysts are presented in Table 1. The photocatalytic activity of the catalysts was estimated for TX and the commercially available wash-up liquid during the photooxidation in the band reactor.

**Table 1:** Physicochemical properties of the support and the photocatalysts

Catalyst	Ti content [wt.%] Surface composition^a^ (a^b^:b^c^)	Dopant content [wt.%] Surface composition^a^ (a^b^:b^c^)	Total surface area *S*_BET_ [m^2^/g]	Mean pore diameter [nm]
Al_2_O_3_	–	–	150.66	8.43
TiO_2_	2.69 (59.92:2.33)	–	149.80	9.62
CIM-Cu	2.62	3.13	128.47	11.07
CIM- Zn	2.34 (61.12:4.51)	2.75 (1.37:2.90)	124.53	11.15
CIM-Co	2.73 (46.73:21.84)	2.69 (0.55:1.78)	129.66	10.75
CIM-Ni	2.79 (51.82:5.07)	2.81 (0.60:1.82)	127.01	11.11
CIM-Fe	2.77 (27.09:11.06)	3.20 (2.60:4.23)	135.69	10.29
CIM-Mo	2.05 (42.97:6.94)	6.30 (7.66:11.85)	131.25	9.74

^a^According to SEM-EDX

^b^Bright points in the SEM image

^c^Dark areas in the SEM image

## Results and Discussion

TiO_2_ (AEROXIDE® TiO_2_ P25*,* Evonik Degussa GmbH formerly Degussa P25), being widely tested (Herrmann 2010) and commercially available, is used as a standard catalyst in various studies, but the application of powder photocatalysts creates great technological problems in recycling, management and disposal. The powdered catalysts are inconvenient to study in the band reactor, as it is a flow-type device. In order to avoid these problems, the supported catalysts were used. The results obtained using suspended and immersed supported TiO_2_ photocatalysts are thought to be very difficult to compare directly.

### Photocatalysts Characterization Results

#### Physicochemical Properties

The physicochemical properties of the catalysts determined by BET and XRF are presented in Table 1. From that data, it is shown that a similar amount of dopant −2.7–3.2 wt.%, was introduced to all photocatalysts, except CIM-Mo. During further impregnation and calcination, the BET surface area changed from 150 to 130 m^2^/g.

**Fig. 1:**
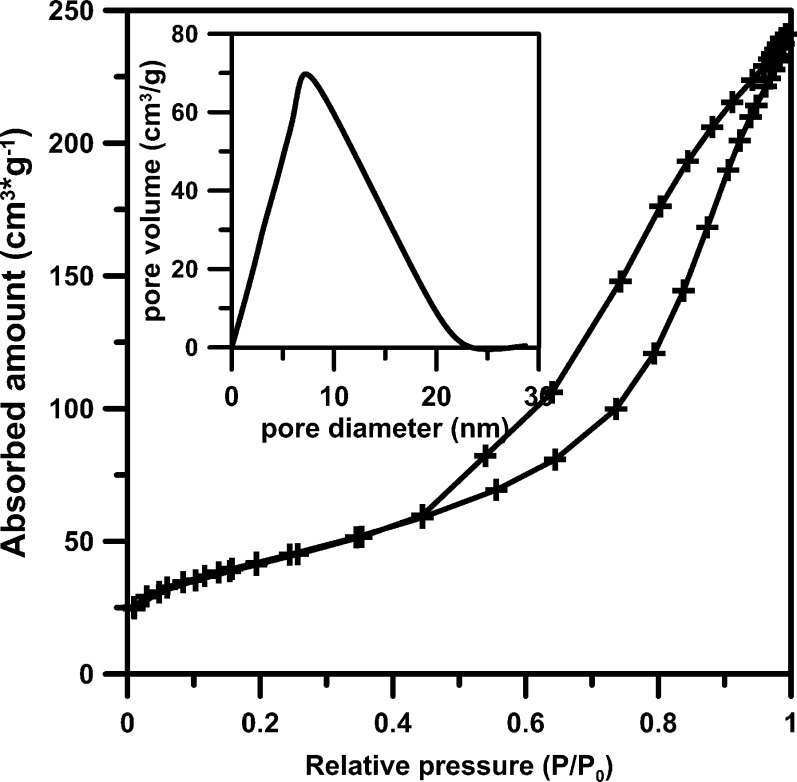
N_2_–adsorption–desorption curves of studied TiO_2_ and pore size distribution in the *inset*

Photocatalytic material is a mesoporous type, which was confirmed by the N_2_-adsorption–desorption studies. All isotherms look similar, and the example TiO_2_ isotherm is presented in Fig. 1. Observed increase in the adsorption branch of the isotherms at a high relative pressure (P/P0 > 0.5) is attributed to uniform pore size distribution (Onsuratoom et al. 2011). The mean pore diameter, 9–11 nm, is similar for all studied photocatalysts and confirms the mesoporosity.

#### SEM and SEM-EDX Studies

**Fig. 2:**
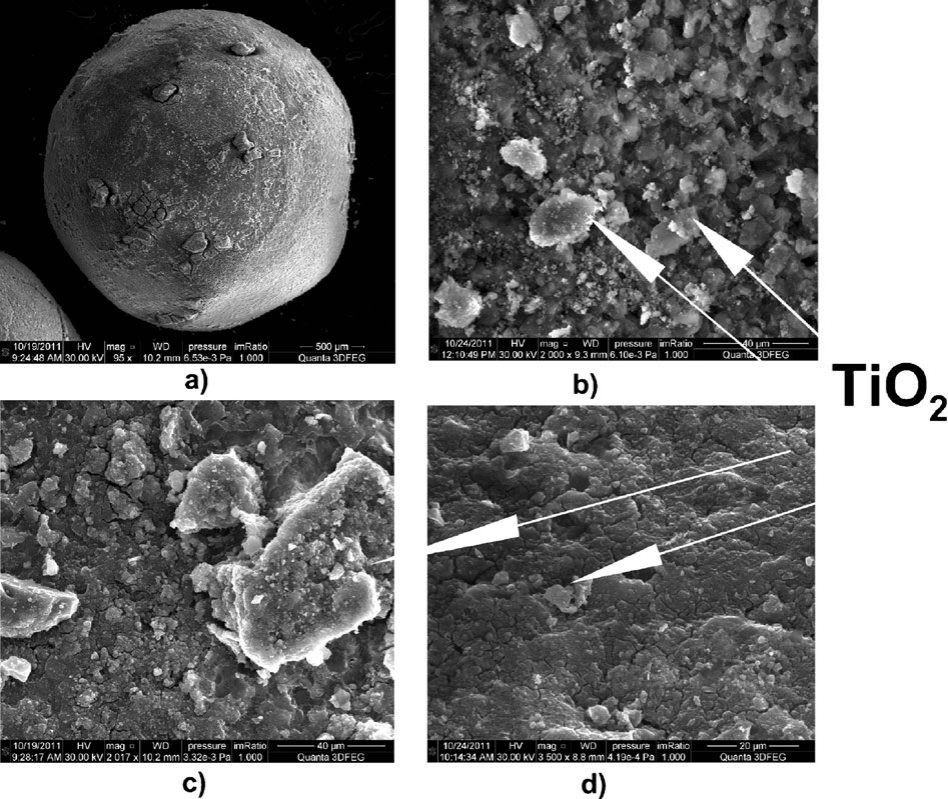
SEM and SEM-EDX images of studied photocatalysts. **a** Bead of CIM-Mo, **b** surface of TiO_2_ /Al_2_O_3_, **c** CIM-Fe, **d** CIM-Co surface

The surface morphology was determined using scanning electron microscopy (SEM) and SEM-energy-dispersive X-ray spectroscopy (EDX). As an example, the micrographs of CIM-Mo, CIM-Fe and CIM-Co are shown in Fig. 2. The presented roughness of the surface of TiO_2_/Al_2_O_3_ beads should ensure better contact of the pollutants and the photocatalysts’ surface. In the SEM micrographs presented in Fig. 2b–d, there are observed bright areas. These points contain generally above 40 wt.% Ti, and some larger crystals have been created. In the case of CIM-Fe, the content of Ti in these points is significantly lower –27 wt.%. On the contrary, Ti content in dark areas is at least two times lower, and on CIM-Mo, CIM-Ni or CIM-Zn surface less than 10 % of Ti is located (Table 1). The dark areas in the SEM micrographs are connected with the presence of Al_2_O_3_ and constitutes of Al, ca. 45–55 wt.% and O 35–40 wt.%. It is evidenced though that TiO_2_ tends to agglomerate over the surface of Al_2_O_3_ beads. SEM micrographs reveal that the TiO_2_ crystals are spherical or oval in shape, and the photocatalysts contained several voids and pores.

The modification of previously prepared TiO_2_/Al_2_O_3_ has resulted in the uniform distribution of dopants. They are present in the same areas of all studied photocatalysts, namely dark points, and their amount in bright and dark areas is remarkably different. They are observed mainly in dark areas with the concentration even threefold increased (for example CIM-Co and CIM-Ni) in comparison with the content in dark areas, which may suggest that their adsorption occurred easier on Al_2_O_3_ than TiO_2_.

#### XRD Studies

**Fig. 3:**
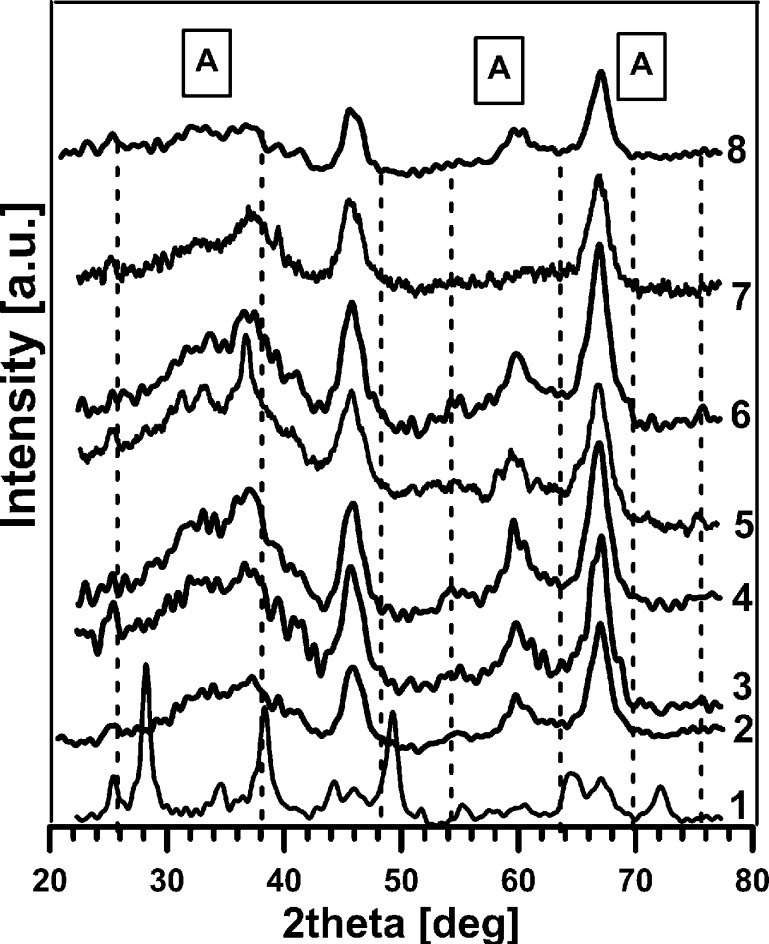
The XRD spectra of the photocatalysts under study: *A*, *broken line*: anatase; *1* Al_2_O_3_, *2* TiO_2_, *3* CIM-Cu, *4* CIM-Zn, *5* CIM-Co, *6* CIM-Ni, *7* CIM-Fe, *8* CIM-Mo

In Fig. 3, there are shown XRD spectra of all studied photocatalysts. All XRD spectra looks similar, and the greatest signal of 2Θ = 66.89 is obtained from the Al_2_O_3_ support (JCPD 01-1303). Present in the XRD spectra are the peaks at 2Θ of about 25.2, 37.9, 48.3, 53.8, 62.7, 68.9, 70.1 and 74.8 which, according to JCPD 21-1272, indicates the presence of an anatase structure (planes 1 0 1, 1 0 3, 2 0 0, 1 0 5, 2 1 3, 1 1 6, 2 2 0 and 2 1 5, respectively). The presence of dopants is not clearly observed, which may indicate the hiding of the signal by the support or the metal dispersion. But the closer analysis of the XRD spectra and JCPD base revealed that there are some observed peaks indicating that Co is present as Co_3_O_4_, Cu as CuO, Zn as ZnO, Ni as NiO, Mo as MoO_3_ and Fe as Fe_2_O_3_. There are also some observed mixed alumina–titania forms.

#### Raman Spectroscopy Results

**Fig. 4:**
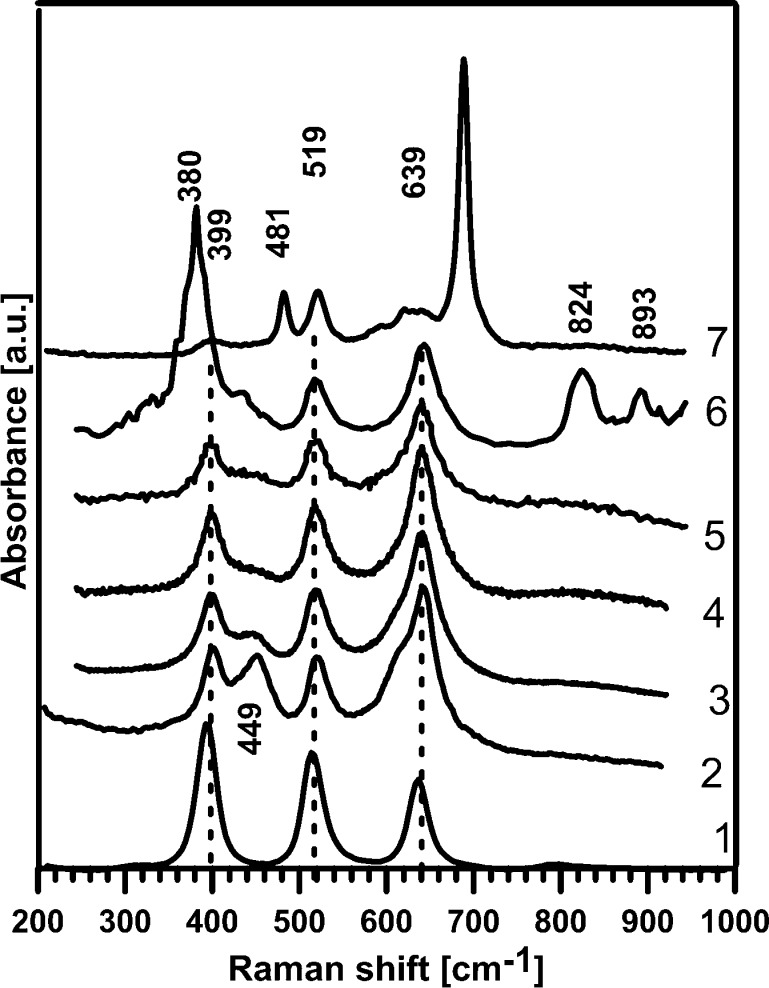
The RAMAN spectra of the studied photocatalysts: *1* anatase, *2* TiO_2_, *3* CIM-Zn, *4* CIM-Ni, *5* CIM-Fe, *6* CIM-Mo, *7* CIM-Co

All studied catalysts were characterized by Raman spectroscopy, and the spectra are shown in Fig. 4. All spectra look similar and peaks by 636, 514 and 396 cm^−1^ (indicated in Fig. 4 by broken lines) are connected with the presence of anatase TiO_2_. Raman modes can be assigned to the Raman spectra of the anatase single crystal: 639 cm^−1^ (*E*_g_), 519 (*B*_1g_), 513 (*A*_1*g*_), 399 (*B*_1g_), 197 (*E*_g_) and ~144 (*E*_g_). The 449-cm^−1^ *A*_1*g*_ and 610-cm^−1^ *E*_*g*_ Raman modes for TiO_2_ in TiO_2_/Al_2_O_3_ decreased during further impregnation and calcination. A slight change in the peak position, line width and shape of the *E*_*g*_ Raman mode in anatase TiO_2_ (e.g. for CIM-Co towards 150, 192, and 651 cm^−1^) may have resulted from phonon confinement, strain, non-homogeneity of the size distribution, defects or non-stoichiometry (Šćepanović et al. 2009).

The characteristic peaks for ZnO, (388, 430, 461 and 498 cm^−1^), NiO (~570, ~730 and ~1,090 cm^−1^) modes are not clearly seen in the picture. The strong signal obtained from anatase in the Raman spectra of all studied catalysts indicates that Zn, Ni or Fe are incorporated into the structure of some alumina or alumina–titania forms. They are probably hidden and superimposed in the spectra though the deconvolution of the peaks was necessary.

The curve-fitting analysis was performed using the Peak Fit programme (Version 4.12). In the Raman spectra of studied photocatalysts, the deconvolution revealed the presence of peaks: CIM-Zn at 447, 597, 680 cm^−1^; CIM-Ni at 212, 325, 453, 583, 691 cm^−1^; CIM-Co at 482, 596, 689 cm^−1^; CIM-Mo at 211, 330, 380, 436, 824, 893, 952, and 1,005 cm^−1^. The peaks in CIM-Mo Raman spectra at 824 cm^−1^ indicate the presence of MoO_3_; in CIM-Co at 689 cm^−1^, CoTiO_3_; and at 496 cm^−1^, Co (Brundle and Morawitz 1982).

The Raman studies results have confirmed the data obtained by SEM/EDX and XRD. The surface of all photocatalysts looks similar. There are some observed agglomerates of TiO_2_, and dopants are introduced and adsorbed over Al_2_O_3_ creating some mixed alumina–titania or alumina–titania–dopant oxides.

#### UV–Visible Spectroscopy Results

**Fig. 5:**
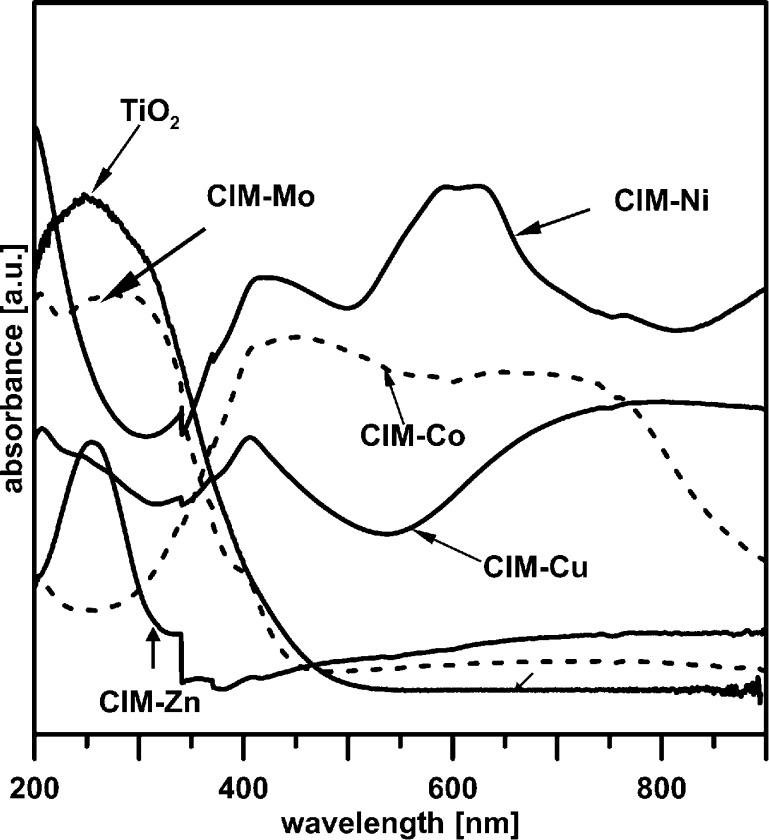
UV–Vis spectra of studied catalysts

In Fig. 5, there are presented UV–Vis spectra of studied catalysts. It can be clearly observed that the absorption band of TiO_2_, CIM-Mo and CIM-Zn is mainly located in the UV light region (200–400 nm). It can be seen that the UV–Vis absorbance for Cu (maximum at 408 nm and over 660 nm), Co (408–481 nm) and Ni (591–629 nm)-modified catalysts shifted to longer wavelengths (the red shift) compared with unmodified photocatalyst.

However, the visible light response of the Cu-, Co- or Ni-modified photocatalysts did not pose any significant effect on their UV photocatalytic activity during the removal of studied pollutants because the employed light source emitted light of more than 95 % within the UV light wavelength but do not exclude their higher activity in Vis.

### Photocatalytic Tests Results

**Fig. 6:**
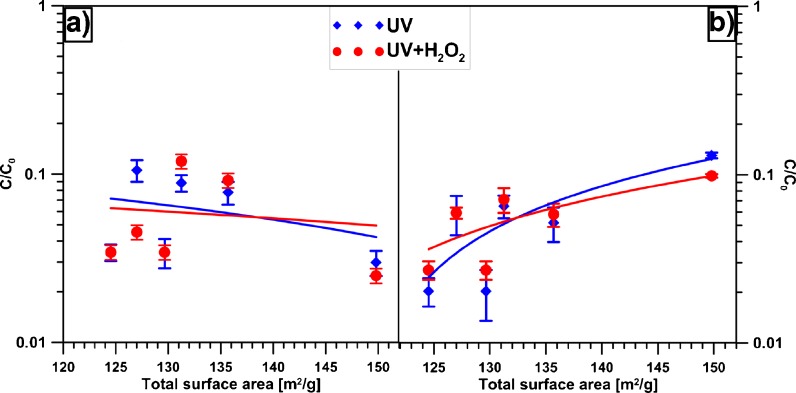
Photoremoval of studied pollutants from wastewater using modified TiO_2_ catalysts **a** TX, **b** WL; *UV* UV irradiation, *UV + H*_*2*_ *O*_*2*_ photooxidation with the addition of H_2_O_2_ in a function of total surface areas of studied photocatalysts

The activity of photocatalysts has been tested in the removal of TX and WL liquid from water. The *c*/*c*_*o*_ has been used to calculate the conversion, where *c*_*o*_ was the initial concentration of a surfactant, and *c* was the concentration of the compounds under study that did not react at a steady state. The best results of TX photocatalytic removal have been obtained using TiO_2_ (Fig. 6a), but the application of CIM-Zn and CIM-Co has also been efficient. The addition of Cu, Ni, Fe and Mo has had the detrimental effect on the TiO_2_/Al_2_O_3_ photocatalytic oxidation. This may suggest that the photoremoval of TX proceeds mainly over TiO_2_.

The correlation between the surface areas of studied photocatalysts and the effectiveness of treatment is presented in Fig. 6. Generally, the removal of TX was independent from the surface area and the observed slight increase towards higher values is, however, not justified economically. The activity of Cu-modified TiO_2_ catalysts is strongly connected with the amount of a dopant by which Cu effectively inhibits the recombination of photo-induced charge carriers. The observed decrease in the removal of TX can be attributed to the excess of Cu in comparison to TiO_2_. It has been stated (Carvalho et al. 2010) that too high Cu values in the photocatalysts result in the more excessive oxygen vacancies, and thus Cu species become the recombination centers of photo-induced e^–^/h^+^. Cu covering the TiO_2_ surface can be noticed. The effect of Ni can be also attributed to the doping type (Yu et al. 2006), since uniform Ni doping can improve the photocatalytic activity only a little.

The synergistic effect of ZnO and TiO_2_ semiconductors has not been observed during the removal of TX. It can be, however, noticed during the removal of more complicated compositions and compounds, e.g. WL (Fig. 6b). That may be in agreement with the literature data, methyl orange photoremoval was slightly enhanced over ZnO-TiO_2_ (Kim et al. 2008).

In the WL removal (Fig. 6b), despite its more complicated composition, deeper photooxidation of organics has been caused by the doping. The effects have been better than over TiO_2_ for all the photocatalysts except CIM-Cu. The effectiveness of removal over CIM-Co or CIM-Zn has been similar—(97 %). It indicates that TiO_2_ may be involved in the oxidation of the non-ionic surfactants, and the dopants have facilitated photooxidation of the other WL components, even inorganic. However, about 5–8 % of the deepened photooxidation using modified photocatalysts has not been economically justified. The decreased efficiency of WL treatment observed for the photocatalysts with enhanced surface area may be connected with the increased adsorption of the components.

The effect of H_2_O_2_ addition has varied depending on the type of the pollutants used, or the photocatalysts (Fig. 6a, b). The photooxidation of TX over TiO_2_ and CIM-Ni has been deepened by H_2_O_2_. Moreover, CIM-Ni seems to be the most sensitive to H_2_O_2_ presence during the removal of TX in the band reactor, as the reduction of COD has increased from about 89 % to 96 %. When it comes to CIM-Co and CIM-Zn, no effect has been observed. For the other photocatalysts, namely CIM-Cu, CIM-Mo and CIM-Fe, the addition of H_2_O_2_ has hindered photooxidation. H_2_O_2_ addition seems not to influence the wash-up liquid photooxidation over any studied catalysts—the obtained results have been almost similar to photocatalytic oxidation.

**Fig. 7:**
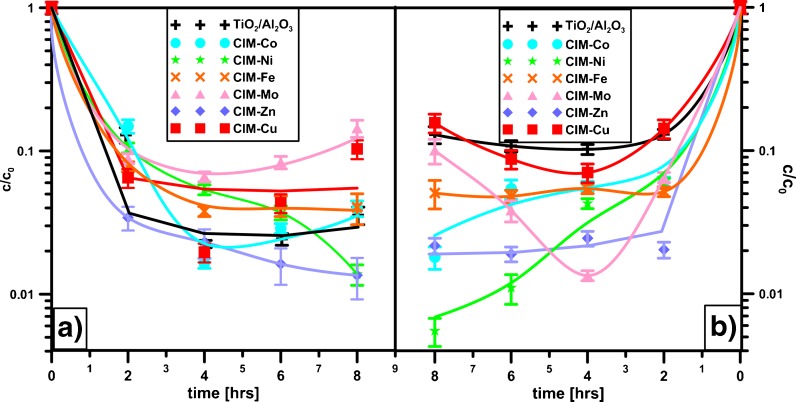
The removal of TX (**a**) and WL (**b**) from wastewater using modified TiO_2_/Al_2_O_3_ catalysts studied in prolonged irradiation time

As the removal of TX and WL was studied in prolonged time, some significant changes have been observed mainly during the first 2 h of treatment. No visible effects in the surfactant removal (Fig. 7a,b) have been caused by time extension. The results obtained over all the photocatalysts have been similar. The desorption of impurities (previously adsorbed surfactant) from the catalysts may slightly affect the effectiveness of the studied reaction—after 4 or 6 h its effectiveness has decreased a bit. The results, however, indicate that for TX removal modification decreases the removal efficiency but in the case of WL, modification of TiO_2_/Al_2_O_3_, especially by Ni, is recommended.

The photooxidation of TX by the UV/TiO_2_ process can be described by the modified Langmuir-Hinshelwood-type kinetic equation. It is assumed that the reaction rate has a linear relation to light intensity for the experiments conducted at constant pH value and TiO_2_ dosage.

For the determination of the kinetics of the removal of TX and WL photooxidation, there was an applied preliminary power law kinetic model (Eq. 1) (Herrmann 2010):

1 $$ r = - \frac{{{\text{d}}c}}{{{\text{d}}t}} = k{c^n}, $$

where *r*, *c* and *t* represents the rate of degradation, pollutants concentration and time respectively. Also, *k* and *n* are the rate constant and reaction order. The rate constants for the apparent consumption of pollutants were obtained from the relation (Eq. 2):

2 $$ - \ln \left( {\frac{\text{COD}}{{\text{COD}}}} \right) = {k_1}t, $$

Where COD_0_ and COD is COD initial and determined at time *t* (minutes), *k*_1_ is the first-order rate constant (per minute). The value *k*_1_ were determined from the slope of the linear dependency, ln(COD/COD_0_) versus *t*. Consequently, half-lives were calculated using Eq. 3.

3 $$ {t_{{1/2}}} = \frac{{0.6931}}{{{k_1}}} $$

The obtained *k*_1_ values changed in the following order (Table 2):

**Table 2:** Pseudo-first-order reaction rate constants (*k*_1_) for photocatalytic oxidation of studied pollutants

Photocatalyst	TX			WL		
	*k*_1_ [×10^−2^ min^−1^]	*T*_1/2_ [min]	*R* ^2^	*k*_1_ [×10^−2^ min^−1^]	*T*_1/2_ [min]	*R* ^2^
TiO_2_	0.9046	76.62	0.763	0.5965	116.19	0.830
CIM-Cu	0.8082	85.75	0.744	0.6114	113.37	0.797
CIM-Zn	0.8256	83.95	0.810	1.0706	64.74	0.834
CIM-Co	1.1244	61.64	0.894	0.9269	74.78	0.911
CIM-Ni	0.9754	71.06	0.960	1.2073	57.41	0.964
CIM-Fe	0.8741	79.29	0.877	0.8289	83.61	0.839
CIM-Mo	0.6405	108.22	0.745	0.8433	82.19	0.731

- TX: CIM-Co > CIM-Ni > TiO_2_ > CIM-Fe > CIM-Zn > CIM-Cu > CIM-Mo
- WL: CIM-Ni > CIM-Zn > CIM-Co > CIM-Mo > CIM-Fe > CIM-Cu > TiO_2_

However, the highest values (over 0.9) of the coefficient of determination (*R* ^2^) were obtained only for Ni-, Fe- or Co-modified photocatalysts which may indicate that only for this photocatalysts the kinetics follows the L-H model.

**Fig. 8:**
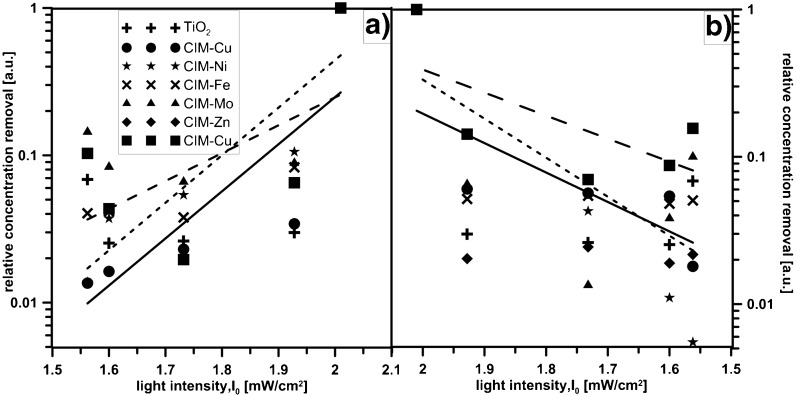
The effectiveness compared with the light intensity **a** TX, **b** WL

The decrease in reaction rates can be attributed to the strong adsorption of organics (Friedmann et al. 2010). Moreover, the UV light intensity in the studied range (1.55–2.01 mW/cm^2^) has enhanced the photooxidation (Fig. 8a,b). The decrease of light intensity below 1.6 mW/cm^2^ may also contribute to the loss of the activity in the COD removal after 6 or 8 h of treatment.

The observed loss of photocatalytic activity during TX removal using the transition metal doped TiO_2_ is suggested to be caused by the increase of the photogenerated e^−^/h^+^ recombination rate (Popa et al. 2010). Therefore, it can be implied that the results of doping catalysts depend on the type of the dopant, its amount and the treated compound.

## Conclusions

The obtained results suggest the following conclusions:

1. All the modified TiO_2_/Al_2_O_3_ photocatalysts are efficient in the removal of the studied pollutants from water;
2. The effect of dopants depends on the type of treated pollutant:
  - The results indicate that the non-ionic surfactants were oxidized mainly by TiO_2_, whereas photooxidation of the other components of the wash-up liquid has been facilitated by dopants;
  - In generally, however, the addition of dopants to the TiO_2_/Al_2_O_3_ during TX and WL removal in the band reactor is not economically justified;
3. The addition of H_2_O_2_ (0.01 vol.%) variously influences the photooxidation of the photocatalysts under study but generally it is not economically justified;
4. Conducting the treatment for more than 2 h is not economically justified.
